# Dual-calibrated fMRI measurement of absolute cerebral metabolic rate of oxygen consumption and effective oxygen diffusivity

**DOI:** 10.1016/j.neuroimage.2018.09.035

**Published:** 2019-01-01

**Authors:** M. Germuska, H.L. Chandler, R.C. Stickland, C. Foster, F. Fasano, T.W. Okell, J. Steventon, V. Tomassini, K. Murphy, R.G. Wise

**Affiliations:** a Cardiff University Brain Research Imaging Centre, School of Psychology, Cardiff University, Cardiff, UK; b Siemens Healthcare Ltd, Frimley, Camberley, UK; c Division of Psychological Medicine and Clinical Neurosciences, Cardiff University School of Medicine, Cardiff, UK; d Wellcome Centre for Integrative Neuroimaging, FMRIB, Nuffield Department of Clinical Neurosciences, University of Oxford, UK

## Abstract

Dual-calibrated fMRI is a multi-parametric technique that allows for the quantification of the resting oxygen extraction fraction (OEF), the absolute rate of cerebral metabolic oxygen consumption (CMRO_2_), cerebral vascular reactivity (CVR) and baseline perfusion (CBF). It combines measurements of arterial spin labelling (ASL) and blood oxygenation level dependent (BOLD) signal changes during hypercapnic and hyperoxic gas challenges. Here we propose an extension to this methodology that permits the simultaneous quantification of the effective oxygen diffusivity of the capillary network (D_C_). The effective oxygen diffusivity has the scope to be an informative biomarker and useful adjunct to CMRO_2_, potentially providing a non-invasive metric of microvascular health, which is known to be disturbed in a range of neurological diseases. We demonstrate the new method in a cohort of healthy volunteers (n = 19) both at rest and during visual stimulation. The effective oxygen diffusivity was found to be highly correlated with CMRO_2_ during rest and activation, consistent with previous PET observations of a strong correlation between metabolic oxygen demand and effective diffusivity. The increase in effective diffusivity during functional activation was found to be consistent with previously reported increases in capillary blood volume, supporting the notion that measured oxygen diffusivity is sensitive to microvascular physiology.

## Highlights

• We propose a new framework for the analysis of calibrated fMRI to map CMRO2 and oxygen diffusivity in the brain.
• The framework is examined with modelling studies and *in-vivo* at rest and during visual stimulation.
• Oxygen diffusivity is found to be highly correlated with CMRO2, evidence of a tight structural-functional relationship.

## 1 Introduction

Calibrated fMRI measurement of absolute cerebral rate of oxygen metabolism (CMRO_2_) offers a non-invasive method of mapping oxygen consumption in the brain (Bulte et al., 2012; Gauthier et al., 2012; Wise et al., 2013), providing quantitative estimates of a critical physiological function. However, the method does not directly consider the transport of oxygen into the tissue, which is principally constrained by cerebral blood flow (CBF) and the effective oxygen diffusivity of the capillaries (Buxton and Frank, 1997; Gjedde et al., 1999; Hayashi et al., 2003; Hyder et al., 1998; Mintun et al., 2001; Vafaee and Gjedde, 2000; Valabregue et al., 2003; Zheng et al., 2002). Effective oxygen diffusivity summarises the practical ability of the capillary network for oxygen diffusion into the tissue and limits the speed of oxygen transport out of the microvasculature. One of the primary determinants of the effective oxygen diffusivity is the capillary density (Gjedde et al., 1999), which is known to be associated with mitochondrial density (Hoppeler and Kayar, 1988) and metabolic demand (Harrison et al., 2002). Thus, brain regions with a high resting CMRO_2_ are found to be co-localised with regions of high capillary density (Sette et al., 1989). However, the effective diffusivity does not appear to be a fixed property of the tissue and may play a crucial role in neurovascular coupling, with oxygen diffusivity being observed to parallel increases in demand and compensate for reductions in oxygen delivery (Hayashi et al., 2003, 2004; Hyder et al., 1998; Vafaee and Gjedde, 2000).

Compartmental models of oxygen exchange between the capillaries and tissue offer a means of estimating the effective oxygen diffusivity from observations of blood flow and oxygen extraction. The model proposed by (Hyder et al., 1998) suggests a need for the effective diffusivity to increase during functional hyperaemia in order to meet the metabolic demands of neural activation. Based on a meta-analysis of CBF and CMRO_2_ measurements from a variety of modalities Hyder et al. proposed a linear coupling between flow and effective diffusivity to account for this apparently coupled behaviour. Evidence for this linear relationship between the effective oxygen diffusivity and CBF was demonstrated with a combined MRI and MRS approach in rat (Hyder et al., 2000). However, PET experiments conducted by (Vafaee and Gjedde, 2000) demonstrate a need for the oxygen diffusivity to adapt to the current metabolic demand, with alterations in the effective diffusivity appearing to be made independently from cerebral blood flow. Alternatively, more recent analysis presented by (Buxton, 2010) demonstrates that metabolic oxygen demand could be met if there was fixed but significant oxygen tissue content, without the need for adjustment to the oxygen diffusivity. The exact mechanism responsible for any such adaptation to metabolic demand is unclear. However, a plausible candidate for the modulation of the effective diffusivity is via pericyte control of capillary dilation, either through a direct increase in the capillary blood volume, or via a homogenisation of flow heterogeneity (Jespersen and Ostergaard, 2012). Thus, measurement of the effective diffusivity may in fact provide a non-invasive probe to investigate the health and action of capillary pericytes, whose function is known to be degraded in multiple neurodegenerative diseases and stroke (Winkler et al., 2013; Yemisci et al., 2009; Zlokovic, 2011).

In the work presented here we use a compartmental model of oxygen exchange to model the relationship between blood flow, effective diffusivity and oxygen extraction. The model is included within a dual-calibrated fMRI estimation framework (Germuska et al., 2016) to enable simultaneous estimates of the resting blood flow, oxygen extraction fraction (OEF), effective diffusivity, and CMRO_2_. The aim of this study was to examine the coupling between consumption (CMRO_2_) and diffusivity at rest, and in response to neural activation (a visual checkerboard task) using the newly proposed method. Our first hypothesis was that, due to the tight functional-structural coupling between capillary density and resting metabolism, there would be a strong correlation between the basal CMRO_2_ and effective diffusivity. Secondly, we hypothesised that the increased metabolic demand due to the visual task would result in a parallel increase in effective diffusivity, whose magnitude should be consistent with published recordings of functional capillary recruitment (Hall et al., 2014).

## 2 Methods

### 2.1 Compartmental modelling

**Fig. 1:**
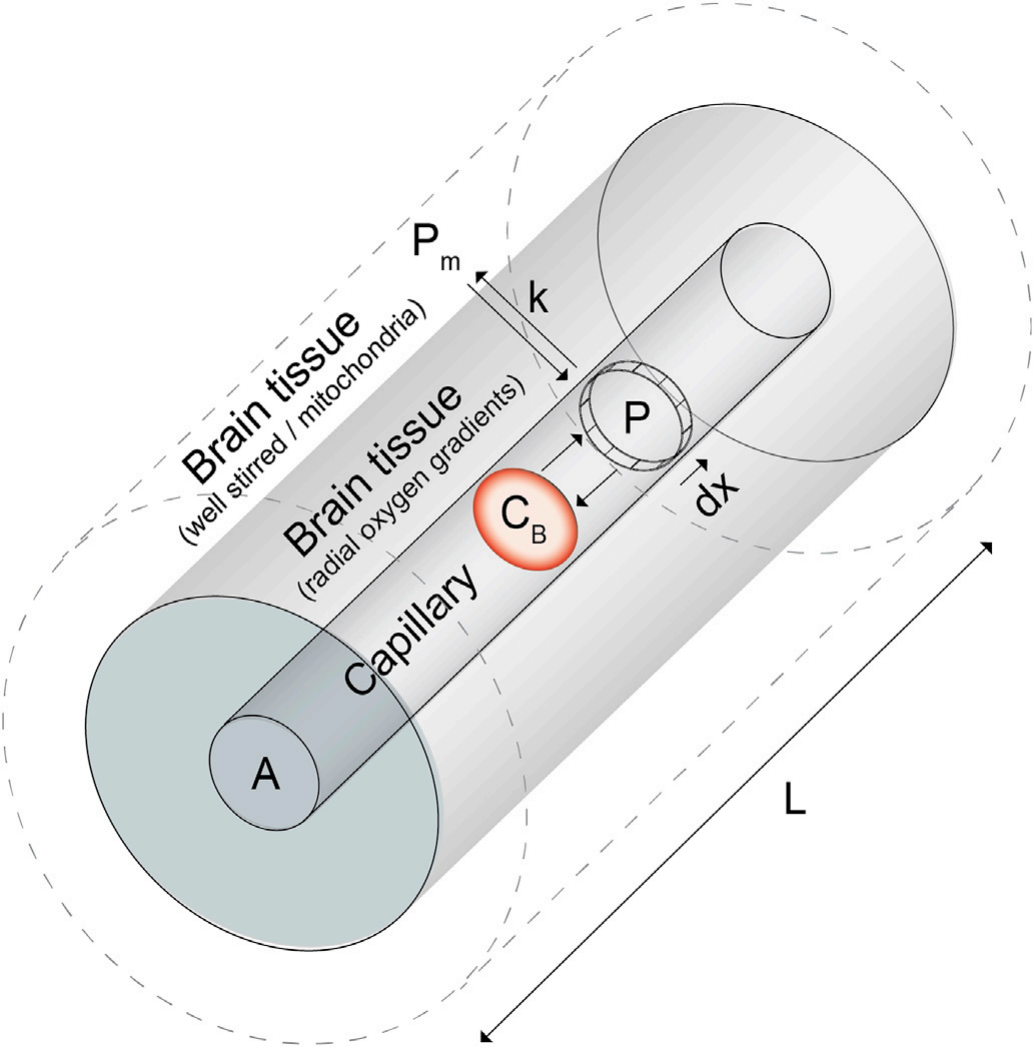
Schematic of the simple compartmental model for oxygen exchange between capillary blood and brain tissue.

The compartmental model of oxygen exchange is based on the model of (Hayashi et al., 2003). As shown in Fig. 1, the model contains a single capillary compartment with area A and length L, which exchanges oxygen with a cylindrical volume of tissue. The capillary has two compartments, a haemoglobin compartment (with oxygen content C_B_) and a plasma compartment (with oxygen partial pressure P). The oxygen in plasma is assumed to be in equilibrium with the oxygen bound to haemoglobin as described by the Hill equation below

(1) P=P50·(CB/(φ·[Hb]−CB))1h

where P is the partial pressure of oxygen in the plasma, P_50_ is the oxygen partial pressure at half saturation, [Hb] is the haemoglobin concentration (g/ml), φ is the oxygen binding capacity for Hb (1.34 ml/g), h is the Hill coefficient (2.8), and C_B_ (oxygen bound to haemoglobin) is equal to total capillary oxygen content, C_t_, if the contribution of plasma oxygen is neglected.

As blood travels along the capillary, the oxygen exchanges between an infinitesimally thin element of blood plasma and a well-stirred oxygen compartment some fixed distance from the capillary (with partial pressure P_m_). The permeability of the capillary endothelium and brain tissue are combined into a single effective permeability, k. This interpretation of the model is a departure from the model presented by (Hayashi et al., 2003), who assumed a uniform partial pressure of oxygen in the radial slice of plasma and zero partial pressure of oxygen on the tissue side of the capillary-tissue interface, thus, localising the oxygen transport to within the capillary endothelium. However, *in-vivo* measurements suggest that the capillary wall does not present a significant barrier to oxygen diffusion (Duling et al., 1979), which is instead provided by the tissue (Hudetz, 1999). Thus, as per (Hyder et al., 1998), we combine both the capillary wall and the surrounding brain tissue into a single interface between the plasma and a well-stirred pool at the end of the diffusion path, which is presumably within or surrounding the mitochondria.

From our compartmental model we can define the differential equation describing the loss of oxygen from within a capillary as

(2) $$\frac{dC_{t}\left(x,t\right)}{dt}=\;-k(P\left(x,t\right)-P_{m}\left(t\right))$$

where t is time, x is the fractional distance along the capillary (0,1), k is the effective permeability (mL/mmHg/mL/min), P is the oxygen partial pressure in the plasma (mmHg), and P_m_ is the oxygen partial pressure at the mitochondria (mmHg).

Following (Zheng et al., 2002) and (Hayashi et al., 2003)

(3) $$CBF=\;A\cdot L\cdot \frac{dx}{dt}=\;V\cdot \frac{dx}{dt}$$

and when blood flow is constant

(4) $$\frac{dC_{t}}{dt}=\;\frac{dC_{t}}{dx}\cdot \frac{dx}{dt}\;$$

where CBF is cerebral blood flow in ml/100 g/min, A is the cross-sectional area of the capillary, L is the length, and V is the volume in ml/100g. Thus, by substituting equations (3), (4) into equation (2) we obtain

(5) $$\frac{dC_{t}}{dx}=\;-\frac{k\cdot V}{CBF}\left(P-P_{m}\right)$$

If we assume that there is minimal partial pressure of oxygen at the mitochondria (Gjedde et al., 1999, 2005; Herman et al., 2006), i.e. P_m_ ≈ 0, then the product k×V is the effective oxygen diffusivity, D_C_ (ml/100g/mmHg/min). By substituting equation (1) into equation (5), we can express the differential equation of oxygen loss in terms of capillary oxygen content, resting CBF, and the effective oxygen diffusivity as

(6) dCtdx=−Dc·P50CBF·(Ctφ·[Hb]−Ct)1h

which is the equivalent to equation (6) in (Hayashi et al., 2003), except that we are assuming negligible oxygen tension at the mitochondria rather than zero average oxygen tension in the tissue.

At the macroscopic level we consider a volume of tissue to contain a collection of identical capillaries arranged such that P_m_ can be considered uniform (note this does not pre-suppose any particular structural configuration). For simplicity we also assume that all other parameters are identical across the capillaries, such that there is no flow heterogeneity or variation in haemoglobin concentration [Hb]. Thus, the modelled oxygen diffusivity represents a combination of vascular parameters including capillary blood volume, flow heterogeneity, and any underlying variation in P_m_.

Equation (6) was solved numerically (using MATLAB's ordinary differential equation solver (Mathworks, MA, USA.)) for different combinations of D_c_, CBF, P_50_, and [Hb] to create a lookup table of results that could be used to fit *in-vivo* data. The oxygen extraction fraction was calculated by evaluating (C_t_(x)|_x=0_ - C_t_(x)|_x=1_)/C_t_(x)|_x=0_ for each combination of parameters, where C_t_(x)|_x=0_ is the oxygen content at the arterial end of the capillary (CaO_2_), assumed to be 0.95 of the maximum (Jespersen and Ostergaard, 2012), and C_t_(x)|_x=1_ is the oxygen content at the venous end of the capillary (CvO_2_).

### 2.2 Calibrated fMRI signal modelling

Quantification of the oxygen extraction fraction and resting blood flow (from which CMRO_2_ is calculated) is performed using the dual-calibrated fMRI method (Bulte et al., 2012; Gauthier et al., 2012; Wise et al., 2013) within a forward modelling framework (Germuska et al., 2016). The method is based upon the isometabolic alteration of flow and venous oxygenation using hypercapnic and hyperoxic respiratory modulations. Here we utilise the simplified calibration model (Merola et al., 2016), where the change in BOLD signal is defined by equation (7).

(7) $$\frac{\Delta S}{S_{0}}=\;TE\cdot \kappa \cdot {\left[dHb\right]}_{0}\left\{1-{\left(\frac{CBF}{CBF_{0}}\right)}^{\theta }\left(\frac{\left[dHb\right]}{{\left[dHb\right]}_{0}}\right)\right\}$$

where S is the MR signal magnitude, TE is the echo time of the acquisition, κ is a composite calibration parameter that represents the combination of the venous-weighted blood volume and water diffusion effects, [dHb] is the deoxyhaemoglobin concentration and is equal to [Hb]×(1-S_v_O_2_), θ (assigned a value of 0.06) is an empirical parameter combining contributions from venous blood volume changes during hypercapnic hyperaemia and extra-vascular water diffusion effects around the microvasculature, and the subscript 0 represents the a parameter's baseline value.

The deoxyhaemoglobin ratio is modelled as shown in equation (8) (Wise et al., 2013), and as before, OEF = (CaO_2_ – CvO_2_)/CaO_2_.

(8) $$\frac{\left[dHb\right]}{{\left[dHb\right]}_{0}}=\;\frac{CBF_{0}}{CBF}-\frac{1}{{\left[dHb\right]}_{0}}\left\{\frac{1}{\varphi }\left(CaO_{2}-\frac{CBF_{0}}{CBF}CaO_{2|0}\right)+\left[Hb\right]\left(\frac{CBF_{0}}{CBF}-1\right)\right\}$$

The arterial spin labelling (ASL) sequence used for the calibrated acquisition uses a pCASL labelling scheme with pre-saturation and background suppression (Okell et al., 2013), and a dual-excitation (DEXI) EPI readout (Schmithorst et al., 2014). As such it differs from the dual-echo PASL acquisition previously employed in the forward modelling framework (Germuska et al., 2016), and the methods have been adapted to reflect this. In the current implementation, BOLD contamination is removed from TE_1_ via surround subtraction, and ASL contamination is removed from TE_2_ via surround averaging. Thus, only the BOLD model (equations (7), (8) is used to estimate TE_2_ data, while TE_1_ time courses are estimated according to the simplified pCASL kinetic model (Alsop et al., 2015), equation (9).

(9) $$\Delta S=\;\frac{2\cdot \alpha \cdot \alpha_{inv}\cdot CBF\cdot T_{1,blood}\cdot M_{0}\cdot \left(1-e^{-\frac{\tau }{T_{1,blood}}}\right)}{6000\cdot \lambda \cdot e^{\frac{PLD}{T_{1,blood}}}}$$

where ΔS is the tag/control difference, α is the tagging inversion efficiency (0.85), α_inv_ is a scaling factor to account for the reduction in tagging efficiency due to background suppression (0.88)(Mutsaerts et al., 2014; Shin et al., 2011), T_1,blood_ is the longitudinal relaxation time of arterial blood, M_0_ is the equilibrium magnetisation, λ is the brain/blood partition coefficient (0.9), τ is the tagging duration, and PLD is the post labelling delay. See Table 1 for a summary of the parameters used in the modelling of ASL, BOLD and oxygen exchange.

**Table 1:** Abbreviations for variables and techniques used in the modelling and analysis.

Variable/abbreviation	Expression (units)
OEF	Oxygen Extraction Faction (dimensionless 0–1)
CMRO_2_	Cerebral Metabolic Rate of Oxygen consumption (μmol/100 g/min)
CBF	Cerebral Blood Flow (ml/100g/mmHg/min)
CVR	Cerebral Vascular Reactivity (% CFB change/mmHg Co_2_)
D_c_	Effective oxygen diffusivity of the capillary network (ml/100g/mmHg/min)
P	Oxygen tension in capillary plasma(mmHg)
P_50_	Oxygen tension at which haemoglobin is 50% saturated (mmHg)
P_m_	Oxygen tension at the mitochondria (mmHg)
[Hb]	Haemoglobin concentration (g/ml)
C_B_	Oxygen content bound to haemoglobin (ml/ml)
C_t_	Total capillary oxygen content (ml/ml)
CaO_2_	Oxygen content at the arterial end of the capillary network (ml/ml)
CvO_2_	Oxygen content at the venous end of the capillary network (ml/ml)
SaO_2_	Arterial oxygen saturation (dimensionless 0–1)
SvO_2_	Venous oxygen saturation (dimensionless 0–1)
PaO_2_	Arterial oxygen tension (mmHg)
PaCO_2_	Arterial carbon dioxide tension (mmHg)
P_ET_O_2_	End-tidal oxygen tension (mmHg)
P_ET-_CO_2_	End-tidal carbon dioxide tension (mmHg)
ϕ	Oxygen binding capacity of haemoglobin (1.34 ml/g)
h	Hill coefficient (2.8)
k	Effective permeability of capillary endothelium and brain tissue (ml/mmHg/ml/min)
ε	Oxygen plasma solubility (0.0031 ml/mmHg/dl)
BOLD	Blood oxygenation level dependent MRI signal
ASL	Arterial spin labelling
TE	Echo time of MRI acquisition (ms)
κ	BOLD calibration parameter including venous-weighted blood volume and water diffusion effects
[dHb]	Deoxyhaemoglobin concentration (g/ml)
θ	Effective hypercapnic venous flow-volume coupling constant (0.06)
T_1,blood_	Longitudinal relaxation time of arterial blood (s)
R_1,blood_	Longitudinal relaxation rate of arterial blood (s^−1^)
M0	MRI signal equilibrium magnetisation (dimensionless)
λ	Brain/blood partition coefficient (dimensionless, 0.9)
τ	Arterial spin labelling tagging duration (s)
PLD	Arterial spin labelling post labelling delay (s)

### 2.3 Data acquisition

**Fig. 2:**
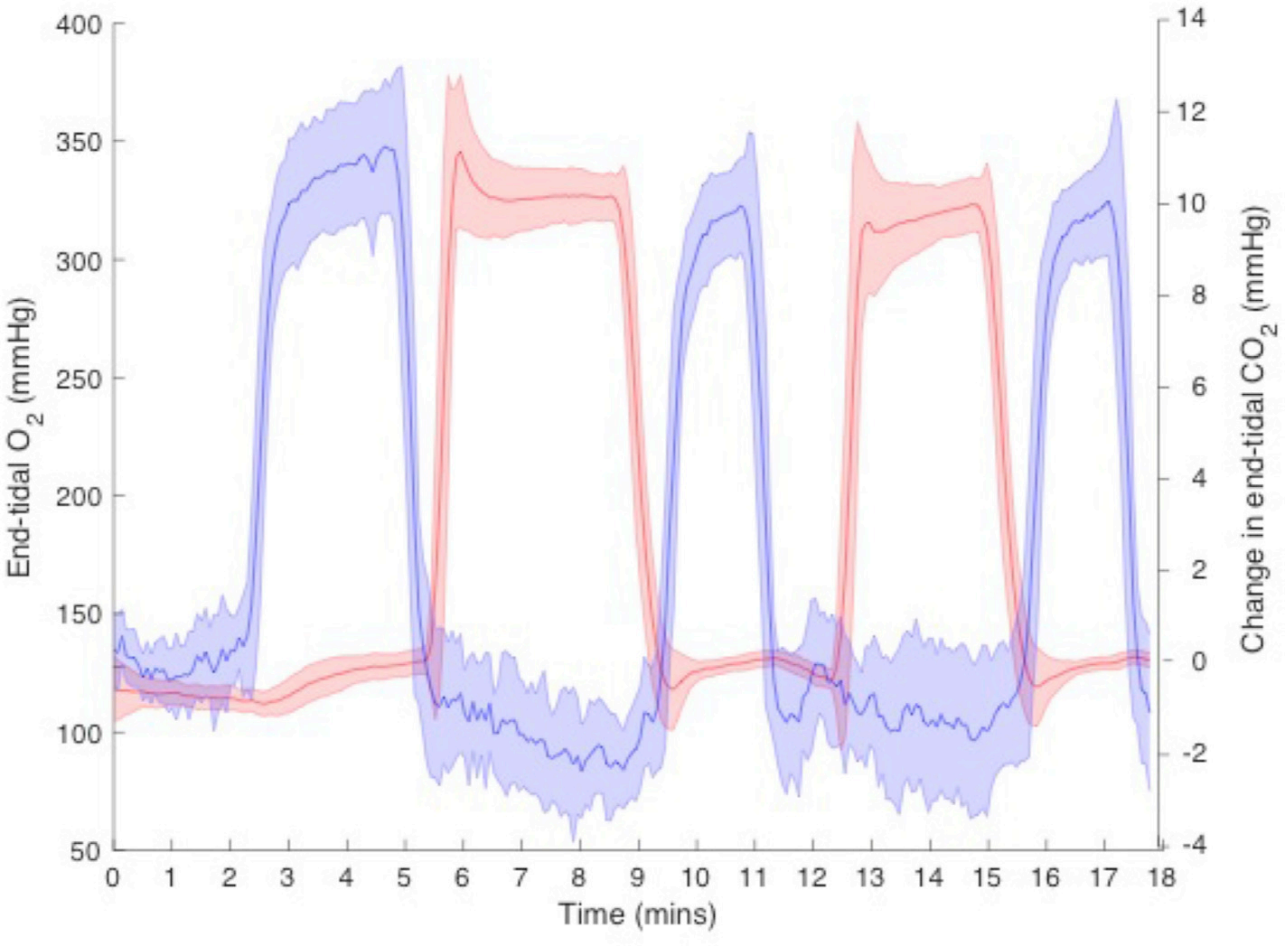
Mean (solid line) and standard deviation (shaded area) of end-tidal recordings from all subjects included in analysis (n = 16). Absolute value of end-tidal oxygen partial pressure (red) and relative change in end-tidal carbon dioxide partial pressure (mmHg).

Nineteen healthy volunteers (13 males, mean age 31.9 ± 6.5 years) were recruited to the study. Volunteers' tolerance of hypercapnic periods and breathing through a face-mask was tested with a non-MRI session prior to MRI scanning. The study was approved by the local ethics committee. Written informed consent was obtained from each participant. All data were acquired using a Siemens MAGNETOM Prisma (Siemens Healthcare GmbH, Erlangen) 3T clinical scanner with a 32-channel receiver head coil (Siemens Healthcare GmbH, Erlangen). During each scanning session an 18-min dual-calibrated fMRI scan was acquired with interleaved periods of hypercapnia, hyperoxia and medical air being delivered to the subjects according to the protocol previously proposed by our lab (Germuska et al., 2016). End-tidal gases, P_ET_CO_2_ and P_ET_O_2_, were sampled from the volunteer's facemask using a rapidly responding gas analyzer (AEI Technologies, Pittsburgh, PA, USA), see Fig. 2 for a summary of end-tidal recordings and timings of the gas paradigm.

**Fig. 3:**
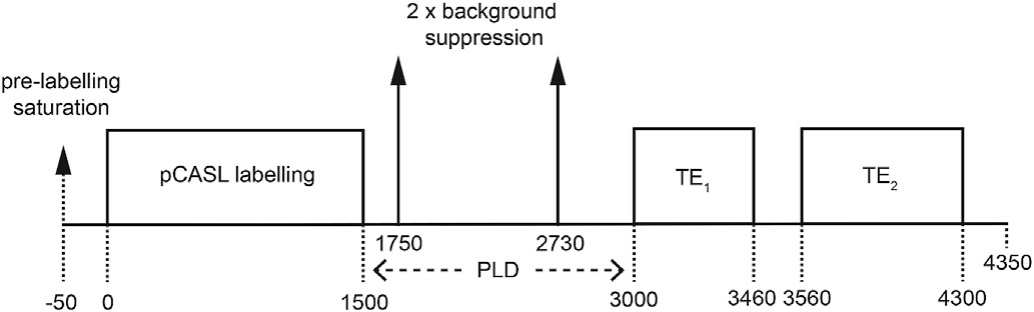
Pulse sequence timing diagram for dual-excitation pseudo-continuous arterial spin labelling (DEXI-pCASL) acquisition. Sequence timings are in ms to the nearest 5 ms.

All calibrated fMRI data were acquired using a prototype pCASL acquisition using pre-saturation and background suppression (Okell et al., 2013) and a dual-excitation (DEXI) readout (Schmithorst et al., 2014), see Fig. 3 for a sequence timing diagram. The labelling duration and PLD were both set to 1.5s, GRAPPA acceleration (factor = 3) was used with TE_1_ = 10 ms and TE_2_ = 30 ms. An effective TR (total repetition time including labelling scheme and both readout periods) of 4.4 s was used to acquire 15 slices, in-plane resolution 3.4 × 3.4 mm and slice thickness 7 mm with a 20% slice gap. A calibration (M_0_) image was acquired for ASL quantification with pCASL and background suppression switched off, with TR of 6 s, and TE = 10 ms.

For a subset of volunteers (n = 7, 3 males, mean age 37.4 ± 6.7 years) an additional 8-min black and white visual checkerboard task (reversing at a frequency of 2Hz, alternating between 30 s rest and 30 s stimulus) was performed during pCASL DEXI data acquisition. In each volunteer a T_1_-weighted structural image was acquired for grey matter segmentation and to aid registration to standard (MNI) space.

Blood samples were drawn via a finger prick prior to scanning and were analysed with the HemoCue Hb 301 System (HemoCue, Ängelholm, Sweden) to calculate the systemic [Hb] value for each participant. The partial pressures of end-tidal gas concentrations were assumed to be in equilibrium with arterial blood, such that PaO_2_ = P_ET_O_2_ and PaCO_2_ = P_ET_CO_2_. Baseline PaCO_2_ recordings were used to estimate resting blood pH based on the Henderson-Hasselbalch equation (equation (10)), assuming HCO_3_- = 24 mmol/L (Gai et al., 2003).

(10) $$pH=\;6.1+log\left\{HCO_{3}^{-}/\left(0.03\cdot PCO_{2}\right)\right\}$$

From which the resting P_50_ was calculated according to the linear correlation, P_50_ = 221.87–26.37×pH, reported by (Gai et al., 2003). The Severinghaus equation (Severinghaus, 1979) was used to convert PaO_2_ recordings into SaO_2_ time series, which were then converted to CaO_2_ via equation (11).

(11) $$CaO_{2}=\;\varphi \cdot \left[Hb\right]\cdot SaO_{2}+\varepsilon \cdot PaO_{2}$$

where ε is the O_2_ plasma solubility (0.0031 ml/mmHg/dL). The T_1_ of arterial blood was calculated from a linear fit to SaO_2_, PaO_2_ and R_1,blood_ in-vivo data presented in (Pilkinton et al., 2012), equation (12).

(12) $$R_{1,blood}=\;1.527\times 10^{-4}\cdot PaO_{2}+0.1713\cdot \left(1-SaO_{2}\right)+0.5848$$

where R_1,blood_ is the longitudinal relaxation rate of arterial blood in seconds.

### 2.4 Data analysis

Data were pre-processed using a combination of MATLAB code and FSL (Jenkinson et al., 2012). Motion correction was performed with the FSL MCFLIRT function and spatial smoothing (FWHM = 4.5 mm) of the BOLD data (surround average of TE_2_) was performed with SUSAN (Smith and Brady, 1997). ASL data (surround subtraction of TE_1_) and M_0_ acquisition were spatially smoothed using a 3D Gaussian kernel (FWHM 4.5 mm). DEXI data was registered to the structural T_1_ data using FSL's epi-reg tool. Following grey matter segmentation of the structural T_1_ image, using FAST (Zhang et al., 2001), grey matter estimates were transformed to native space and used for grey matter masking (threshold of 0.5). DEXI data was masked prior to analysis using a binarised M_0_ image to reduce processing time.

**Fig. 4:**
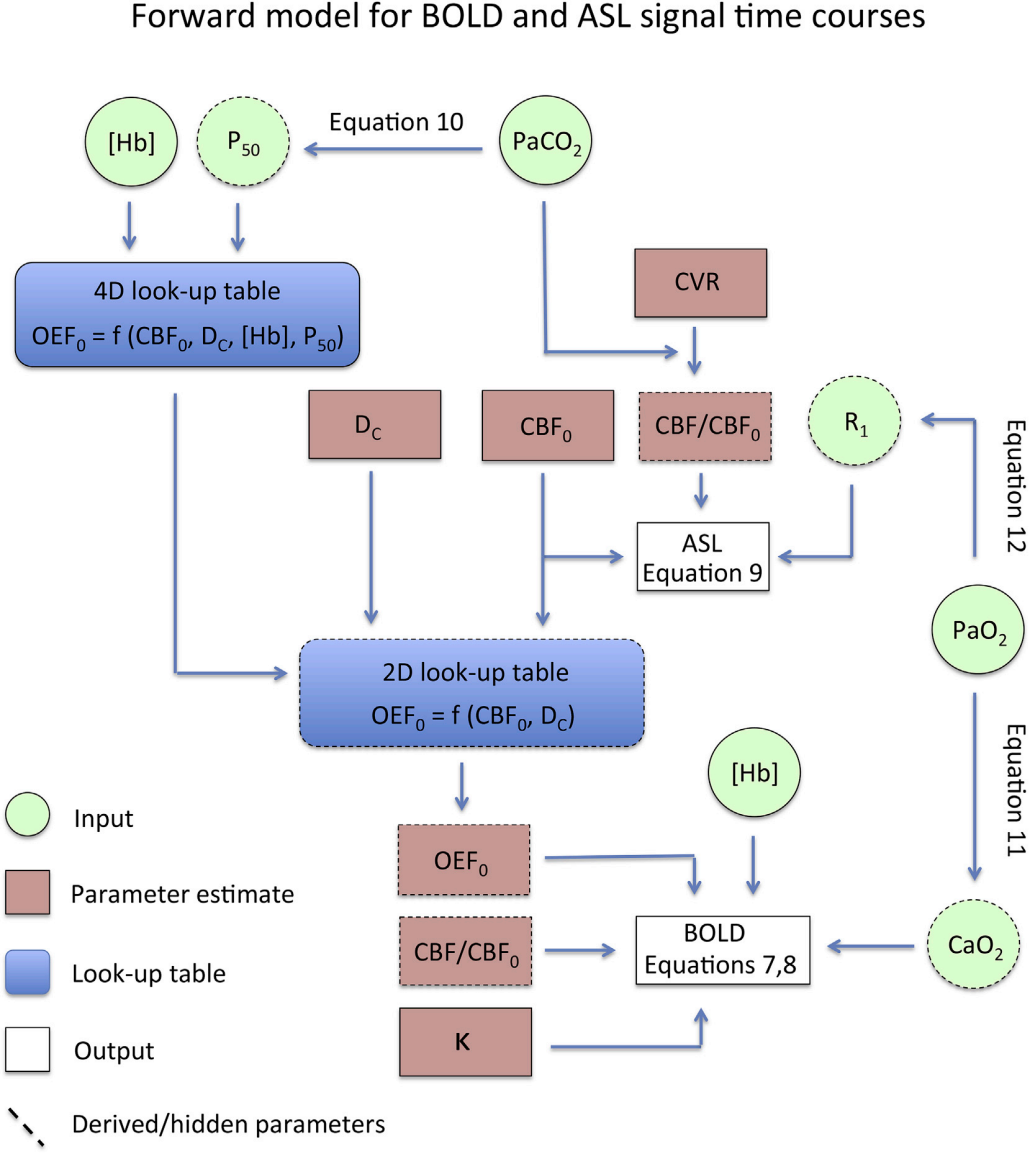
Flow diagram showing how measured physiological data and estimated parameters are combined to estimate ASL and BOLD signal time courses during parameter estimation. The forward model incorporates oxygen diffusivity modelling into a dual-calibrated fMRI framework.

End-tidal traces were aligned with the DEXI data via a cross-correlation between PaCO_2_ and the mean grey matter ASL signal. Measured [Hb] and calculated P_50_ values were used to resample the initial 4D effective diffusivity lookup table to a high-resolution 2D lookup table relating CBF_0_ and D_c_ to OEF, enabling simple linear interpolation to be used during fitting. MATLAB's non-linear least squares minimisation routine (lsqnonlin) was used to simultaneously optimise voxelwise estimates of D_c_, OEF_0_, CBF_0_, κ, and the cerebral vascular reactivity (CVR) by minimising the least squares difference between the acquired data and modelled ASL and BOLD timeseries (MATLAB code for pre-processing of end-tidal traces and parameter estimation is available from 10.5281/zenodo.1285862 and 10.5281/zenodo.1285845). See Fig. 4 for a flow diagram representing the forward model used in the analysis framework. In line with the forward modelling approach previously published (Germuska et al., 2016) regularisation was applied to reduce instability in fitting a non-linear model to the data (see appendix for full details). Briefly, regularisation was applied to D_C_ and OEF in an adaptive manner to reduce the sensitivity to noise variation across voxels and subjects. The regularisation parameter for oxygen extraction fraction was assumed to be uniform, with a nominal OEF_0_ of 0.4. We make the assumption that oxygen diffusivity varies with capillary density (and therefore grey matter partial volume) to impose spatial variation on the diffusivity regularisation. Grey matter partial volume estimates were calculated by normalising an initial perfusion estimate by its maximum value (median value in 100 voxels with greatest signal intensity) and then multiplying by 0.15 ml/100g/mmHg/min. We use an initial perfusion estimate (rather than a segmented structural image) to estimate grey matter partial volume to avoid bias due to segmentation and registration errors. As per our previous work (Germuska et al., 2016), we used digital phantom experiments to determine the optimal level of regularisation for each parameter, OEF and D_C_. Additionally, we explored the influence of the SNR on the mean squared error of regularised fits to the simulated data; see the appendix for further details of the simulations.

Visual data was subject to the same pre-processing steps as the baseline data, additionally it was registered (FLIRT) to baseline data to account for any gross subject motion between the datasets. Percentage change in CBF and the BOLD signals were calculated using FEAT to fit the pre-processed data with the visual paradigm. CMRO_2_ was calculated both on a voxel-wise basis, and from a grey matter ROI, which was thresholded to include only voxels with significant BOLD and CBF activation (z-stats > 2.3). Because resting data was quantified using a simplified BOLD model (Merola et al., 2016), the standard calibration model (Davis et al., 1998) was modified as per equation (13) to calculate the visual CMRO_2_.

(13) $$CMRO_{2}=CMRO_{2,0}\cdot \;\left(1-\frac{\frac{\Delta BOLD}{BOLD_{0}}}{TE\cdot \kappa \cdot {\left[dHb\right]}_{0}}\right)\cdot {\left(\frac{CBF}{CBF_{0}}\right)}^{1-\theta }$$

Estimates of CMRO_2_ and CBF during visual activation were used to calculate OEF via the Fick principle, while estimates of D_C_ were made by inverting the look-up table and assuming constant [Hb] and P_50_.

## 3 Results

The mean and standard deviation of baseline P_ET_O_2_ and P_ET_CO_2_ were 116 ± 5 mmHg and 41.6 ± 3.6 mmHg, the hyperoxic respiratory modulation resulted in an average P_ET_O_2_ of 325.2 ± 12.8 mmHg, while hypercapnia produced an average P_ET_CO_2_ of 51.7 ± 3.5 mmHg. Three subjects did not return to a stable P_ET_CO_2_ baseline during the medical air periods of the DEXI acquisition; this was judged to be a deviation of greater than 4 mmHg below the starting value. These subjects were excluded from further analysis. The mean increase in grey matter CBF during hypercapnia was 24.0 ± 3.7%. Group average values of the resting grey matter physiological parameters are reported in Table 2.

**Table 2:** Mean (±standard deviation) systemic and grey matter estimates at baseline, (n = 16).

[Hb] g/dl	P_50_ mmHg	CBF ml/100 g/min	OEF	CMRO_2_ μmol/100 g/min	D_C_ ml/100g/mmHg/min	CVR %/mmHg
14.3 ± 1.5	27.1 ± 0.1	55.6 ± 6.3	0.38 ± 0.04	157.4 ± 12.3	0.092 ± 0.009	2.4 ± 0.4

**Fig. 5:**
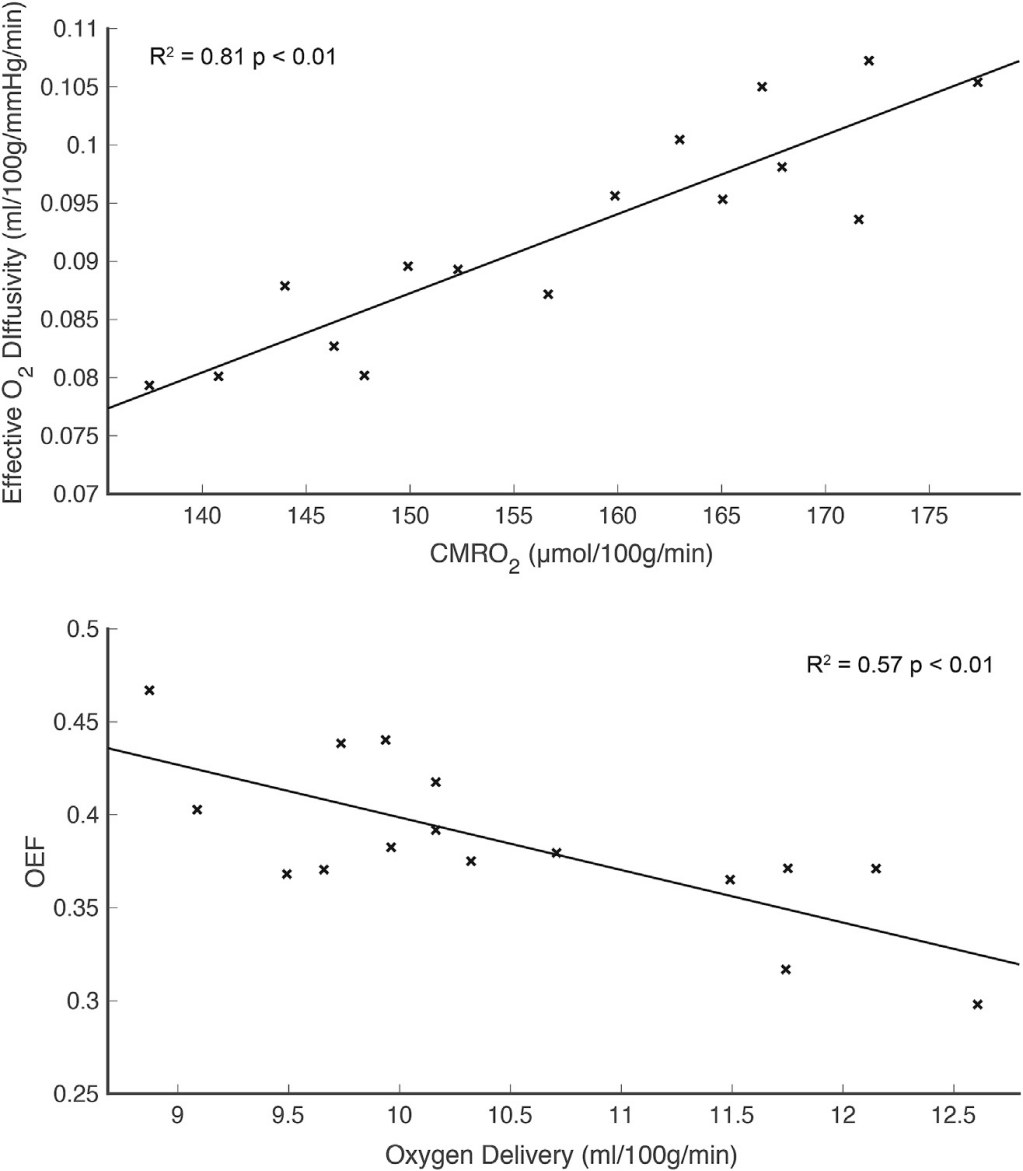
Scatter plots of whole brain grey matter parameter estimates at baseline for each subject (n = 16). Top panel (A) demonstrates a strong correlation between baseline metabolic oxygen consumption and effective oxygen diffusivity. Bottom panel (B) shows a strong negative correlation between oxygen extraction and oxygen delivery, such that delivery is elevated when OEF is low.

**Fig. 6:**
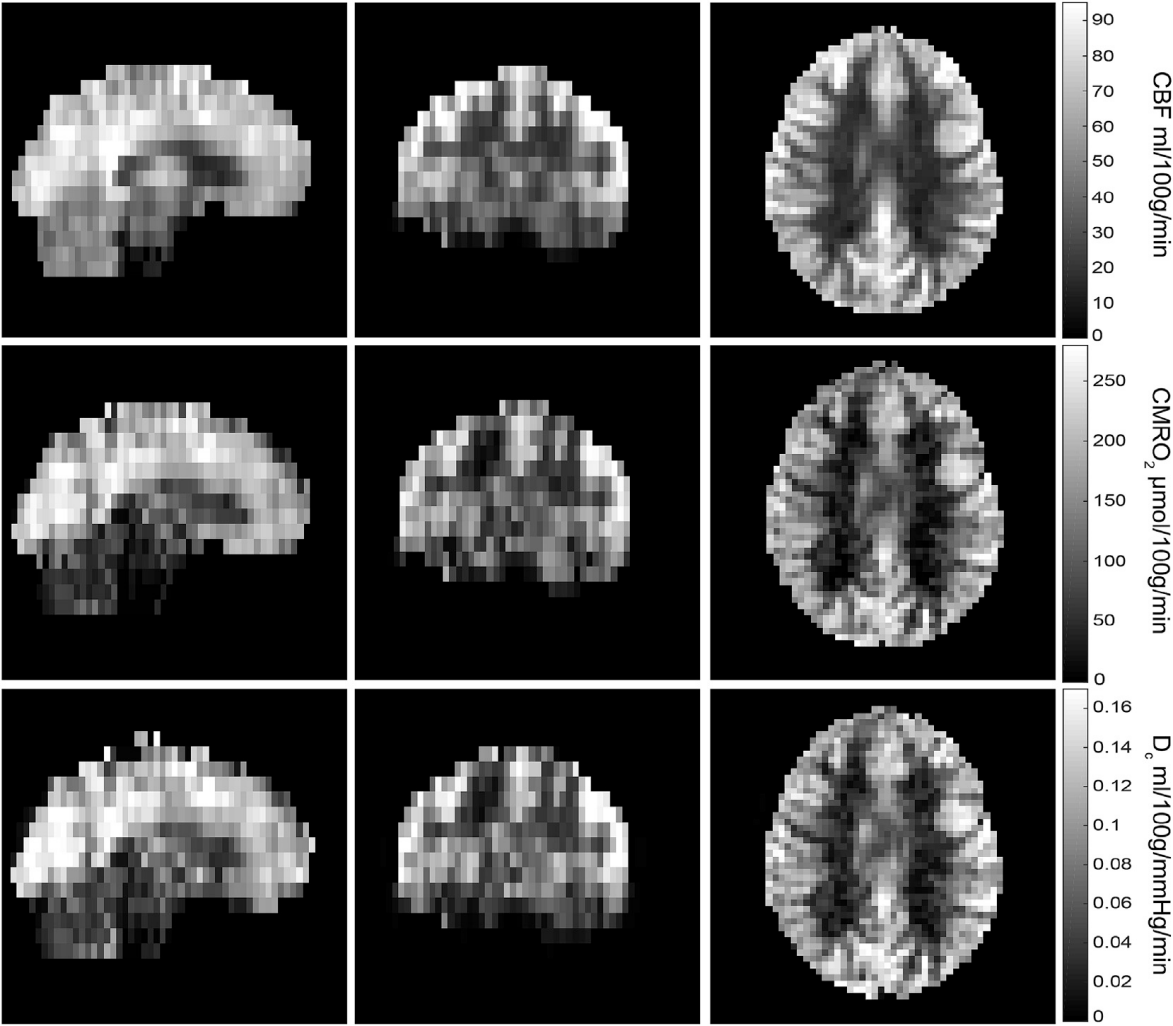
Example baseline (CBF_0_, CMRO_2,0_ and D_c,0_) parameter maps for an individual subject. The spatial similarity between oxygen diffusivity and the basal rate of oxygen metabolism is evidence of a strong structural-functional coupling between the two parameters in the basal state.

The mean grey matter value of the effective diffusivity was 0.092 ± 0.01 ml/100g/mmHg/min, or 3.62 ± 0.39 μmol/100g/mmHg/min, which is in good agreement with the PET measurements made by (Ibaraki et al., 2010) and (Vafaee and Gjedde, 2000) who report value of 3.38 and 4.09 respectively. Baseline values of CMRO_2_ are strongly correlated with the effective oxygen diffusivity (R^2^ = 0.81, p < 0.01), across participants, as shown in Fig. 5a. This coupling between CMRO_2_ and oxygen diffusivity is also demonstrated within subjects, with a clear spatial similarity between the baseline parameter maps. Example resting parameter maps (CBF_0_, CMRO_2,0_ and D_c,0_) for an individual subject are shown in Fig. 6. The similarity between the parameter maps is clear, with the maps of effective diffusivity closely following CMRO_2_.

While the effective diffusivity appears to be strongly coupled to resting demand, we observed a strong negative correlation between baseline oxygen delivery (CaO_2_×CBF) and oxygen extraction (R^2^ = 0.57, p < 0.01) (Fig. 5b). This is consistent with previous findings of a strong negative correlation between OEF_0_ and CBF_0_ (Germuska et al., 2016; Lu et al., 2008) when [Hb] was assumed to be constant. By incorporating a measured [Hb] in the calculation of CaO_2_, the correlation with oxygen delivery is revealed; demonstrating the action of the cerebrovascular system to maintain a tightly controlled resting metabolic rate of oxygen consumption across participants.

**Fig. 7:**
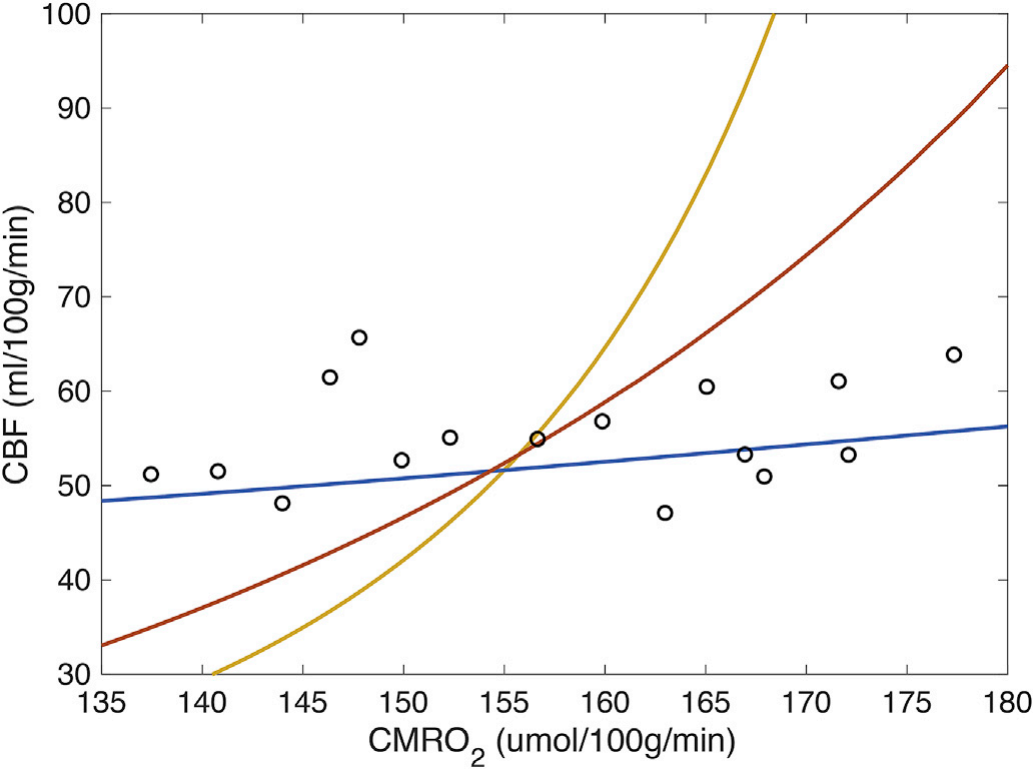
Modelled relationship between CMRO_2,0_ and CBF_0_ for linear increase in D_C,0_ with CMRO_2,0_ (blue), constant D_C,0_ (orange), and linear decrease in D_C,0_ with CMRO_2,0_ (yellow). In-vivo CMRO_2,0_ and normalised CBF_0_ (CaO_2,0_⋅CBF_0_/0.189), mean grey matter values overlaid (circles).

To investigate the observed linear relationship between CMRO_2,0_ and D_C,0_, we solved the inverse model with fixed [Hb] (14.3 g/dl), P_50_ (27.1 mmHg), and CaO_2,0_ (0.189 ml O2/ml blood). By doing so we are able to examine the modelled co-variance between CMRO_2,0_ and CBF_0_ in isolation of other physiological factors, see Fig. 7. The analysis suggests a small positive correlation between CBF_0_ and CMRO_2,0_ is required to produce a strictly linear relationship between CMRO_2,0_ and D_C,0_, which agrees well with the in-vivo data (p < 0.05 for a paired *t*-test comparison between the predicted CBF_0_ and the normalised CBF_0_ estimates (CaO_2,0_ x (CBF_0_/0.189)). However, if the effective oxygen diffusivity is considered to be a constant physiological parameter, as is sometimes assumed (Vafaee et al., 2012), then a significant non-linear (exponential) relationship is predicted between CBF_0_ and CMRO_2,0_. Finally, we explored a hypothetical scenario in which the inverse of the correlation observed in this study exists between CMRO_2,0_ and D_C,0_. This scenario requires a much larger increase in CBF_0_ with CMRO_2,0_, but it is still mathematically and physiologically feasible to produce such a relationship. This analysis highlights the fact that even though D_C,0_ and CMRO_2,0_ appear to be coupled (in the examined cohort of young healthy volunteers) they provide complementary information, with CMRO_2_ reporting on the rate of oxygen consumption and D_C_ reporting on the ability of the capillary network to supply oxygen to the tissue/mitochondria. The coupling between these parameters is likely the result of the expected correlation between microvascular structure (diffusivity) and function (metabolism).

**Fig. 8:**
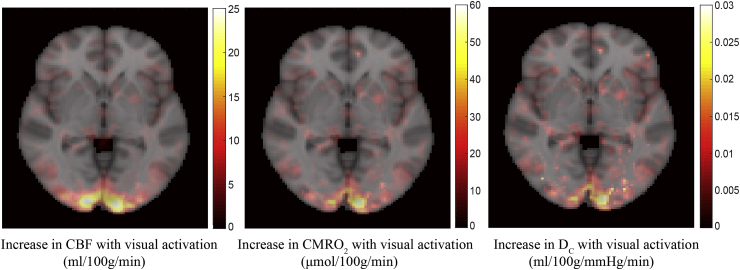
Overlay of the mean absolute change in CBF, CMRO_2_, and effective oxygen diffusivity evoked by the visual checkerboard stimulus for n = 7 subjects.

During visual simulation cerebral blood flow increased within the defined ROI (in the primary visual cortex, as expected) by 21.4 ± 4.6%, while CMRO_2_ increased by 15.1 ± 3.5%, resulting in a CBF to CMRO_2_ coupling ratio of 1.44 ± 0.24. The effective oxygen diffusivity was found to increase by 12.5 ± 3.6%. The CMRO_2_ and diffusivity increases are of a similar magnitude to those observed with PET measurements using a 4Hz yellow-blue contrast-reversing checkerboard, where CMRO_2_ and oxygen diffusivity were found to increase by 14.9% and 9.0% respectively. Fig. 8 shows the mean (n = 7) absolute change in physiological parameters evoked by the visual stimulus overlaid onto the selected slice in standard (MNI) space. Changes in diffusivity are co-localised with changes in CMRO_2_, whereas changes in CBF are more widespread.

**Fig. 9:**
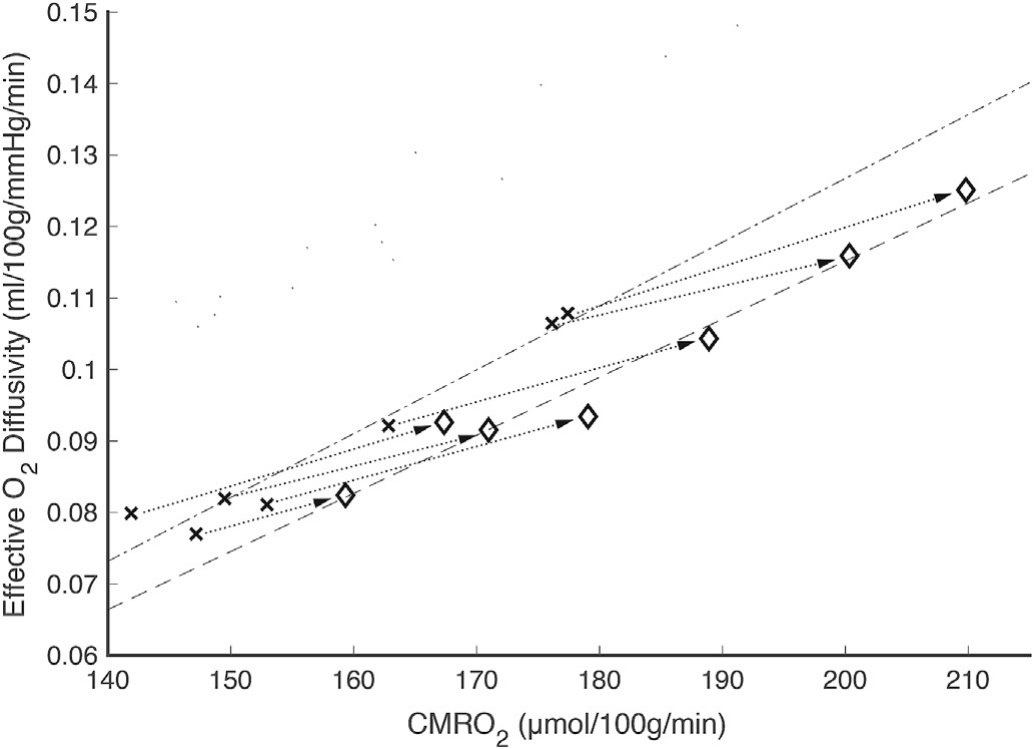
Summary plot of visual ROI data for CMRO_2_ and effective oxygen diffusivity (including resting and activation data for n = 7 subjects). A tight correlation between CMRO_2_ and effective diffusivity is observed both at baseline (crosses) and during activation (diamonds), indicative of a tight coupling between the effective diffusivity and oxygen demand. The dashed lines are lines of best fit (linear regression) the dotted lines connect baseline and activation data for each subject.

Fig. 9 shows the correlation between the average CMRO_2_ and effective diffusivity within the visual ROI at baseline (crosses) and during activation (diamonds) for each participant. It is clear from the graph that the coupling between demand and diffusivity, that was previously observed in the grey matter baseline data, is preserved during activation, R^2^ (baseline) = 0.95, R^2^ (activation) = 0.96, p < 0.01 for both datasets.

Under the assumption that P_m_ is minimal and the effective permeability is held constant during activation, the apparent change in capillary blood volume is linearly related to the effective diffusivity (see equations (5), (6)). Thus, we can calculate the apparent flow-volume coupling relationship, CBV/CBV_0_ = (CBF/CBF_0_)^η^, implied from the diffusivity data. Using this line of reasoning we find that a coupling constant of 0.62 ± 0.13 is required, relating the 21% flow change to an apparent 12.5% volume increase. This result agrees surprisingly well with in-vivo observations of functional capillary vasodilation. Where the 6.7% increase in capillary diameter observed by (Hall et al., 2014) was calculated to produce a 19% increase in blood flow according to Poiseuille's law. Assuming constant capillary length this observation would predict a flow-volume coupling exponent of approximately 0.75 for their data.

## 4 Discussion and conclusions

In this manuscript we have presented a novel framework for the analysis of dual-calibrated data to produce simultaneous estimates of CMRO_2_ and effective oxygen diffusivity. The combined analysis of these two physiological parameters has the potential to provide useful insight into the underlying metabolic and vascular responses to different brain states and disease. The method was applied at rest and in combination with a visual task. The resting data showed a tight coupling between grey matter diffusivity and the basal rate of oxygen metabolism. This result is expected in the healthy brain, as there is significant evidence of a structural link between the density of capillaries (a significant determinant of the effective diffusivity) and metabolism (Gjedde et al., 1990; Harrison et al., 2002; Sette et al., 1989).

The effective diffusivity was also found to increase during functional activation, with a 12.5% increase in diffusivity being associated with a 15.1% increase in CMRO_2_ and 21.4% increase in CBF. The coupling ratio between CBF and CMRO_2_, 1.44, is at the lower end of *in-vivo* observations, which typically range from 1.3 to 5 for MRI methodologies (Leithner and Royl, 2014). Thus, the change in effective diffusivity is likely to be at the higher end of the expected range (in order to meet the oxygen demands of the elevated CMRO_2_ in the absence of a greater increase in CBF). Possible mechanisms to provide such an increase in effective diffusivity include a direct increase in capillary blood volume (Hyder et al., 1998), a homogenisation of capillary flow heterogeneity (Jespersen and Ostergaard, 2012), a reduction in the mitochondrial oxygen tension (Gjedde et al., 2005), or a high resting tissue oxygen tension (Buxton, 2010). While each of these factors could play a role in modulating the diffusivity of the capillary network, *in-vivo* measurements suggest that tissue oxygenation initially increases during functional activation and then normalises to a level slightly above its resting value (Ances et al., 2001). Thus, it is unlikely that there is a significant reduction in mitochondrial oxygen tension, which would be expected to lower tissue oxygenation rather than increase it. Alternatively, a high resting mitochondrial oxygen tension would result in a small vessel-to-tissue PO_2_ gradient, which, as highlighted by (Hyder et al., 2000), increases the effectiveness by which CBF can increase O_2_ delivery to the tissue. Our model predicts that the mitochondrial oxygen tension would need to be unrealistically high, approximately 30 mmHg to explain the CBF/CMRO_2_ increases observed in this study. While there is uncertainty in the value of P_m_ in the human brain, animal studies suggest that it is between 0.1 and 10 mmHg (Herman et al., 2006), thus a significant resting mitochondrial oxygen tension is unlikely. An alternative explanation explored in this paper is that there is a direct increase in capillary blood volume, potentially mediated by capillary pericytes; which have been demonstrated to alter capillary volume independently of arteriolar dilation (Mishra et al., 2016) and appear to play a significant role in neurovascular coupling (Kisler et al., 2017). For a given rate of perfusion, an increase in capillary volume would increase the mean transit time for blood to transverse the capillary network, producing a proportional increase in the effective diffusivity, and thus enabling greater extraction of the oxygen from the capillary bed. Our data suggest that a flow-volume coupling exponent of approximately 0.62 is required in the capillaries to provide the observed increase in effective diffusivity during visual stimulation. The implied 12.5% increase in capillary blood volume agrees well with the data and analysis presented by (Hall et al., 2014) in the mouse brain, suggesting this is a plausible explanation for the observed increase in diffusivity. Indeed more recent studies in the mouse brain suggest that such an increase in capillary blood volume is well within the range of normal physiological responses, where capillary volume has been found to increase by 10–26% during functional activation depending on the baseline diameter (Ito et al., 2017).

Although modulation of capillary blood volume appears to be sufficient to provide local control of capillary diffusivity, we cannot rule out a contribution from flow heterogeneity (Jespersen and Ostergaard, 2012), which is likely to be reduced during functional activation where smaller capillaries have been observed to dilate more than larger capillaries in rat (Kleinfeld et al., 1998; Stefanovic et al., 2008). However, the influence of flow heterogeneity is known to be dependent on the transit time distribution (Angleys et al., 2015), and confounding factors such as the heterogeneity of [Hb] have not been considered in the modelling, thus it is still unclear if this theoretical model of control is realised *in-vivo*.

As previously discussed, there is unlikely to be a significant resting P_m_ in the studied cohort, meaning the assumption of negligible P_m_ is unlikely to impact the results. However, this may not always be true. In theory P_m_ could increase in the presence of significant mitochondrial dysfunction due to the lack of oxygen uptake in the mitochondria. Currently the only direct evidence we are aware of for an increase in tissue (and therefore most likely mitochondrial) oxygen tension during mitochondrial dysfunction is from simulated dysfunction (due to a cyanide infusion) in piglets (Nielsen et al., 2013). However, there is increasing evidence of mitochondrial dysfunction in a number of neurodegenerative diseases such as Parkinson's disease (Powers et al., 2008) and Alzheimer's disease (Wang et al., 2014). Therefore, it should be highlighted that reductions in basal D_C_ may not always correspond to a purely vascular origin and may also incorporate mitochondrial dysfunction.

In line with our previously published methods for analysis of dual-calibrated data (Germuska et al., 2016; Wise et al., 2013) we have included priors to stabilise the fitting process (see appendix). In the current implementation the priors are incorporated into parameter estimates via adaptive regularisation. Whenever priors are used to guide the fitting process there is always a trade off to be made between overfitting (not enough regularisation) and underfitting (too much regularisation). Digital phantom simulations were used to optimise the amount of regularisation and balance this trade-off. The proposed method employs regularisation on two parameters, the resting OEF and the effective diffusivity. In the case of underfitting we would expect the results to closely follow the prior, thus there would be little variation in OEF or the effective diffusivity between subjects. In contrast we find that OEF is highly correlated with resting oxygen delivery, and the effective diffusivity is tightly coupled to the resting CMRO_2_. Thus, it is unlikely that the results are significantly affected by underfitting. While we cannot rule out overfitting of the data, the appearance of the parameter maps is physiologically plausible and they do not suffer from significant instability within the grey matter. To further explore the influence of the proposed framework on the parameter estimates we performed simulations where D_C_ was not estimated. In this implementation OEF was estimated directly as in (Germuska et al., 2016), and thus no regularisation was/could be placed on D_C_. These simulations (see appendix) showed similar levels of error to the proposed method, albeit with slightly increase RMSE in OEF estimates at low tSNR.

In conclusion, we have presented an MRI method for mapping the effective oxygen diffusivity of the capillary bed in combination with metabolic oxygen consumption. The method shows good agreement with PET literature and inferred changes in capillary blood volume are in agreement with two-photon laser microscopy measurements in animals, however, direct validation of the method is still outstanding. The proposed method is non-invasive and can be performed in a short timeframe. Previous measurements of effective oxygen diffusivity suggest it may be a valuable tool to understand the brain's response to altered oxygen supply and demand. Thus, the introduction of this method could offer a useful insight into a range of conditions and diseases with altered metabolism or vascular function.

## Figures and Tables

**Fig. A1:**
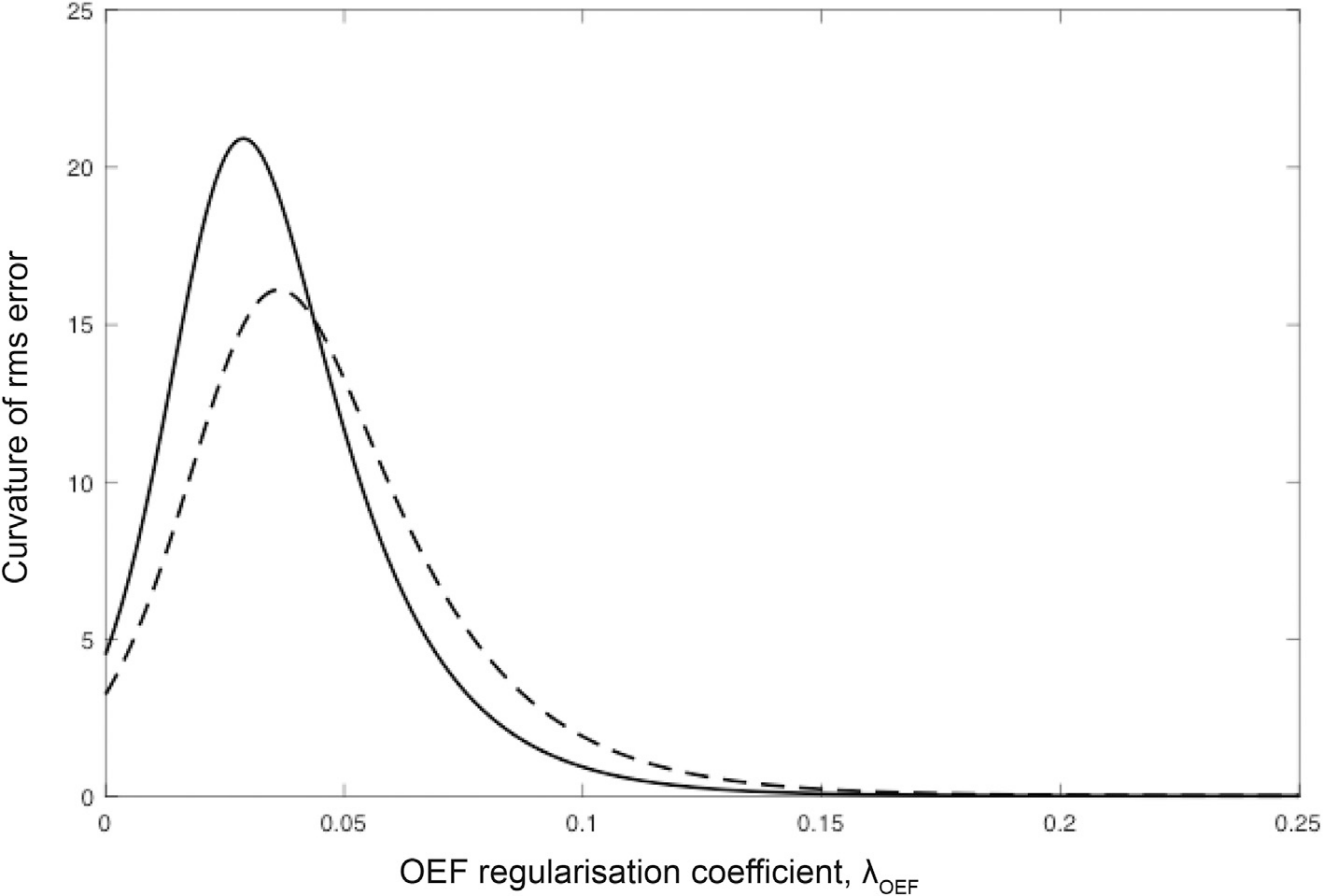
Plot of λ_OEF_ vs. curvature of rms error for effective diffusivity (solid line) and OEF (dashed line). The curvature of the diffusivity error reaches a maximum before the curvature of the OEF error, therefore this point (0.03) was chosen for lambda.

**Fig. A2:**
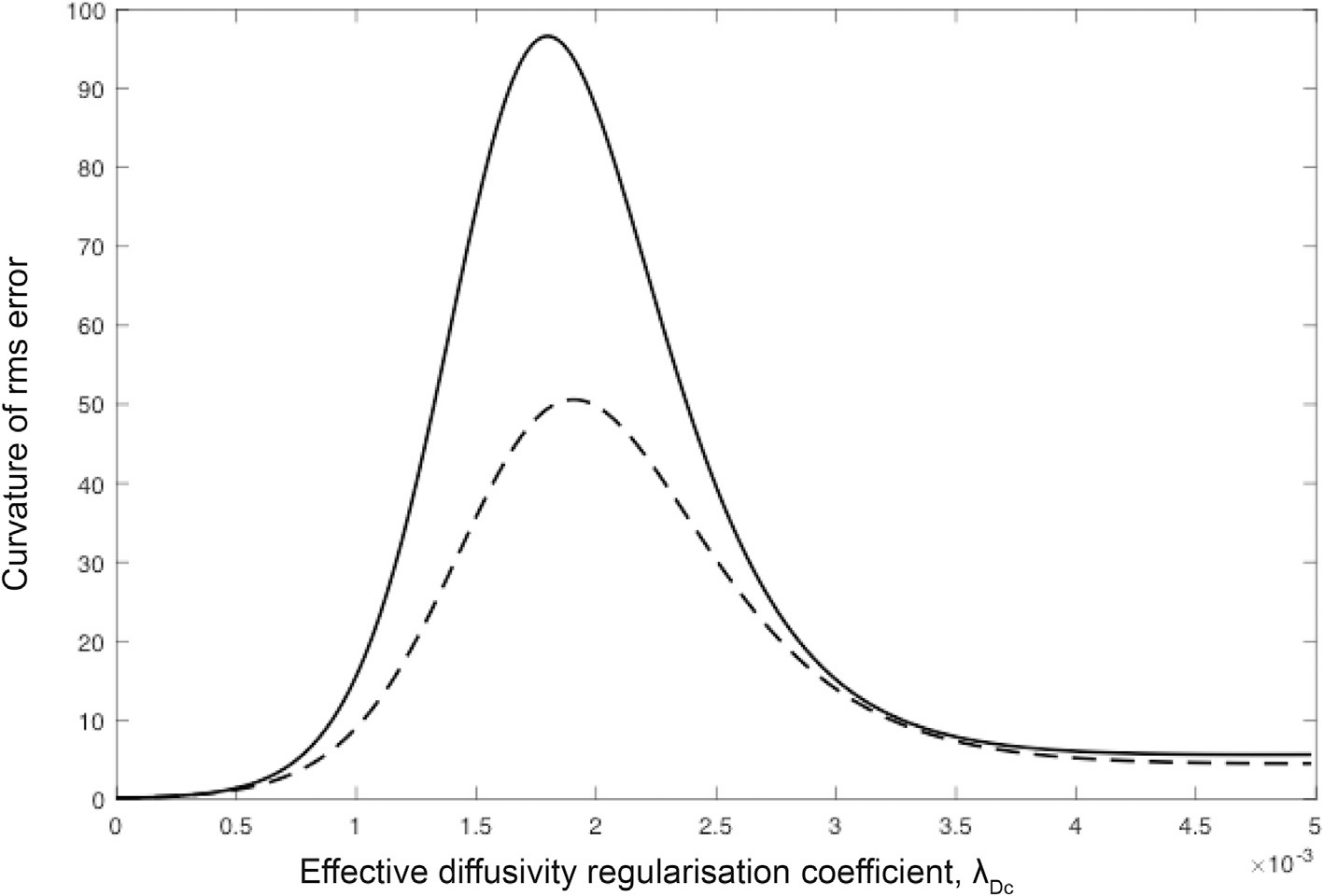
Plot of λ_D_c vs. curvature of rms error for effective diffusivity (solid line) and OEF (dashed line). The curvature of the diffusivity error reaches a maximum marginally before the curvature of the OEF error. The peak of the D_C_ curvature is at approximately 1.8 × 10^−3^, and therefore this value is used to set the level of regularisation for D_C_ estimates.

**Fig. A3:**
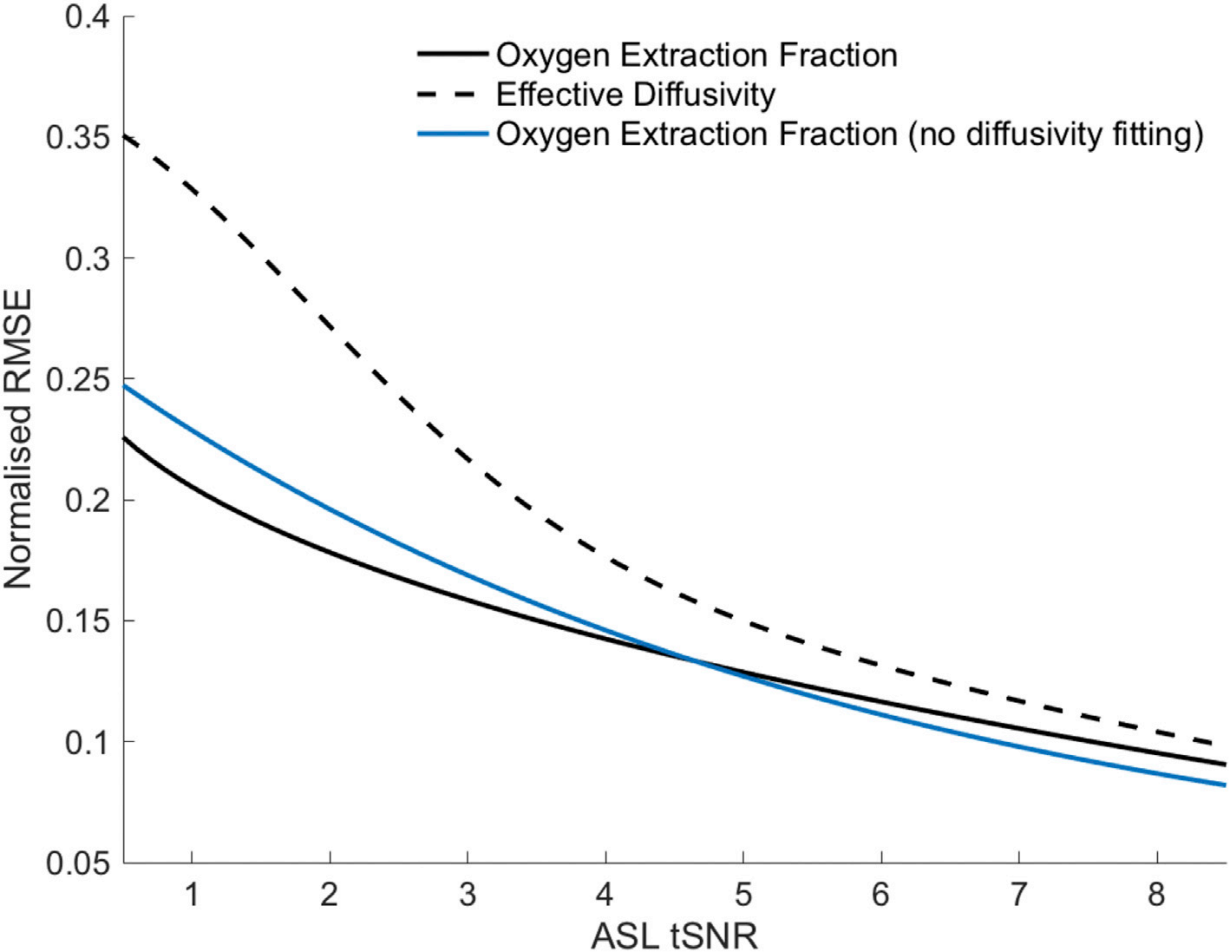
Plot of normalised RMSE in OEF and D_C_ predicted with varying levels of tSNR (ASL) from digital phantom simulations.

## Appendix Digital phantom simulations

We can model the observed MRI data, ASL and BOLD signals that we wish to fit, via equation (A1).

(A1) $$Y_{i}=f\left(w_{i},X\right)+e_{i},\;e∼N\left(0,\sigma^{2}\right)$$

Where Y represents the observed MRI signal data, X represents the corresponding physiological traces and their derived parameters (see Fig. 2), w is a vector of physiological parameters, e is signal noise with zero mean and an unknown variance σ^2^, and *i* is the voxel index.

In order to estimate the physiological parameters, *w*, we perform regularised non-linear least squares minimisation (via MATLAB's lsqnonlin function), as defined by equation (A2).

(A2) $$w_{i|RLS}=\min_{w}\left(∥f\left(w_{i},X\right)-Y_{i}{∥}_{2}^{2}+\lambda {\hat{\sigma }}_{i}^{2}∥w_{i}^{'}-v_{i}{∥}_{2}^{2}\right)$$

Where w_i|RLS_ is the regularised least squares estimate of the physiological parameters at the *i*th voxel. λ is a vector of regularisation coefficient, ${\hat{\sigma }}^{2}$ is a voxelwise estimate of noise variance (calculated from the variance of the residuals), w’ is a vector comprising the parameter estimates for D_C_ and OEF, and v is a vector of the corresponding expected values. The expected value for OEF is set to 0.4 for all voxels (representing a nominal OEF near the mid-point of the physiological range), while the expected value for diffusivity estimates are linearly related to voxel-wise grey matter partial volume estimates. In this way we impose a prior belief that the effective diffusivity will be linearly related to grey matter content (due to the associated relationship with CBV). The expected value for pure grey matter voxels is set to a nominal D_C_ value of 0.15 ml/100g/mmHg/min, corresponding to an OEF of 0.35 (Ibaraki et al., 2008), when Hb = 0.15 g/ml, P_50_ = 26 mmHg, and CBF = 90 ml/100 g/min (Asllani et al., 2008).

Because the noise variance is estimated on a voxel-wise basis the regularisation is spatially adaptive; with greater regularisation applied when the noise variance estimate is greater. It should also be noted that the magnitude of the regularisation reduces as the minimisation routine approaches the solution, which has been shown to reduce the bias at convergence (Hong et al., 2017). The value of the regularisation coefficients λ_Dc_ and λ_OEF_, which define the vector λ, were determined from digital phantom simulations, as described below.

Simulated phantoms were created with 245 time points (matching the in-vivo acquisition), for both the ASL and BOLD data, and 4200 elements. Effective diffusivity values were randomly chosen in the range 0.03–0.18, OEF values were assigned values from 0.25 to 0.55, Hb was set to 0.15 g/ml, P_50_ was set to 26 mmHg, if any combination of values implied a CBF below 20 or above 150 ml/100 g/min (according to the flow-diffusion modelling) another value was randomly chosen until this criteria was met. The tSNR of the simulated data was set to match that of the acquired data (after spatial smoothing), which were approximately 4.5 for the ASL data and 150 for the BOLD data. In order to match to in-vivo noise statistics, simulated ASL and BOLD time series were band-pass filtered with a second order infinite impulse response filter using the MATLAB ‘designfilt’ function. The relative bandpass frequencies were 0.08–0.2 for the ASL data and 0.01 to 0.2 for the BOLD data (pass band ripple 1 dB in each case).

The RMS error in OEF and D_C_ estimates were calculated for varying levels of regularisation under two conditions; with regularisation applied only to OEF and with regularisation applied only to D_C_. Thus, optimising the required regularisation for each parameter separately. By analogy to the standard L-curve method (Hansen, 1992), the optimal level of regularisation was chosen to be the maximum of the curvature of the λ vs RMS error plot (see figures A1 and A2). Thus, selecting the transition point above which further regularisation ceases to provide significant reductions in RMS error. Because we are optimising the regularisation for two parameters (and in two conditions), there are two transition points to choose between in each condition. To minimise the chances of underfitting we chose the point that corresponds to the smallest amount of regularisation in each case.

Having optimised λ for a fixed tSNR we explored the noise sensitivity of OEF and D_C_ estimates for ASL tSNR ranging from 0.5 to 8.5 (BOLD tSNR was linearly scaled with ASL tSNR and so ranged from 17 to 280). The results of these simulations are shown in figure A3 and demonstrate a non-linear increase N-RMSE for both OEF and D_C_ as the tSNR is reduced. It is apparent from the simulation that there is less uncertainty in OEF estimates, with a 15% error occurring for a tSNR of approximately 3, while D_C_ requires a tSNR of approximately 5 to achieve the same level of uncertainty. For comparison with previous methods, which do not fit for D_C_, we have also included the error in OEF estimates when fitting for OEF directly. This is equivalent to the implementation in (Germuska et al., 2016), but with adaptive (rather than fixed) regularisation placed only on OEF. The regularisation for this approach was tuned to match the current implementation when the ASL tSNR = 4.5. As can be seen from the plot, the error in OEF estimates are similar to the newly proposed method, with a slight reduction for high tSNR and a decrease in performance for low tSNR. This decrease in performance for low tSNR is likely due to the convergence of the fit to a uniform prior, rather than a prior informed by the estimate of baseline CBF.
